# Consumer Consciousness in Multisensory Extended Reality

**DOI:** 10.3389/fpsyg.2022.851753

**Published:** 2022-04-21

**Authors:** Olivia Petit, Carlos Velasco, Qian Janice Wang, Charles Spence

**Affiliations:** ^1^Kedge Business School, Department of Marketing, Marketing and New Consumption Centre of Excellence, Marseille, France; ^2^BI Norwegian Business School, Department of Marketing, Centre for Multisensory Marketing, Oslo, Norway; ^3^Department of Food Science, Faculty of Technical Sciences, Aarhus University, Aarhus, Denmark; ^4^Department of Experimental Psychology, University of Oxford, Oxford, United Kingdom

**Keywords:** consciousness, consumer behaviour, virtual reality, augmented reality, extended reality, metaverse, multisensory experience

## Abstract

The reality-virtuality continuum encompasses a multitude of objects, events and environments ranging from real-world multisensory inputs to interactive multisensory virtual simulators, in which sensory integration can involve very different combinations of both physical and digital inputs. These different ways of stimulating the senses can affect the consumer’s consciousness, potentially altering their judgements and behaviours. In this perspective paper, we explore how technologies such as Augmented Reality (AR) and Virtual Reality (VR) can, by generating and modifying the human sensorium, act on consumer consciousness. We discuss the potential impact of this altered consciousness for consumer behaviour while, at the same time, considering how it may pave the way for further research.

## Introduction

Mark Zuckerberg, recently changed the name of Facebook to ‘Meta’, indicating that metaverse will be one of the key future directions of the Internet. It is estimated that in 2026, 25% of people worldwide will spend at least 1 h a day in the metaverse for digital activities, including shopping, social interaction and entertainment (Johnson, 2022). The growing enthusiasm for the metaverse suggests that consumers will increasingly accept a dematerialization of their extended-self, leading them to ascribe greater value to virtual objects (Belk, 2013; Velasco et al., 2021b). However, will these possessions necessarily be similar to those that exist in the physical world? Is the dematerialization of our extended-self in the metaverse not likely to affect the way in which we make decisions? It is clearly going to be important for forward-thinking brands to question the impact of the metaverse on consumer behaviour.

In the metaverse, consumers will likely be able to live and share their experiences in a way that will more-or-less closely resemble their experiences in real-life (albeit with a much-reduced sensory bandwidth, catering primarily to the visual and auditory senses). AR, VR and other visual-enabling technologies already allow the users to navigate and interact with people and objects virtually (Flavián et al., 2019) and stimulate the senses in real-time (Ueda et al., 2020; Barbosa Escobar et al., 2021; Wang et al., 2021). These technologies have been used extensively during the COVID-19 outbreak, helping people overcome the isolation, and currently continue to develop (Kerdvibulvech and Chen, 2020; Kerdvibulvech and Dong, 2021). In addition, the latest (multi-)sensory-enabling technologies (SETs, e.g. mid-air haptics and smell interfaces) will certainly be integrated in the metaverse to stimulate the consumer’s senses directly (Petit et al., 2019; Velasco and Obrist, 2020). These technologies will thus provide sensory immersive experiences close to consumers’ everyday offline experiences (Spence and Gallace, 2011; Petit et al., 2019). However, in the metaverse, these sensory inputs can be limited, amplified, made congruent or incongruent with the digital and/or physical environment in which the user finds themselves. Since the way we become conscious of our surroundings and our bodies depends largely on our senses (Ionta et al., 2011; Nani et al., 2019), the question is, how can their stimulation in the metaverse affect the way we make decisions? Thus, it becomes essential to understand how people make predictions on the basis of sensory inputs when engaged in impression formation, when they involve both virtual and physical objects and environments.

## The Role of the Human Senses in Consumer Consciousness

Consciousness can be understood as ‘*a controlled hallucination based on predictions about the current sensory inputs’* Metzinger (2018, p. 4). Many researchers working in the neurosciences have highlighted the key role of sensory inputs, and recurrent feedback, in the establishment/maintenance of consciousness (Dehaene and Naccache, 2001; Baars, 2002; Gallace and Spence, 2008). Conscious visual experience is thought to emerge from the processing and transmission of information from sensory areas up to higher-order cortical and motor regions (Nani et al., 2019; Sikkens et al., 2019). In turn, the temporo-parietal occipital workspace is continuously scanned and accessed by the attention network. While bottom-up subcortical mechanisms support wakefulness, top-down cortical mechanisms are important as far as delivering the contents of consciousness is concerned (Mashour and Hudetz, 2017). Thus, consciousness depends on whether a stimulus is perceptible and how, exactly, attention is deployed.

In the context of consumer behaviour, consciousness also plays a crucial role (Plassmann and Mormann, 2017; Williams and Poehlman, 2017). According to Baumeister et al. (2017), consumer consciousness contributes to choices and wellbeing notably by aligning consumer behaviour with the self. Consumer consciousness can take various forms with different impacts on consumer behaviour. For example, consumer health consciousness has been shown to have a positive effect on the intention to purchase organic food products (Nagaraj, 2021). Body consciousness has also been shown to impact how women imagine they can control their physical appearance, leading them to invest time in creating self-portraits to express their identity in social media (Boursier et al., 2020).

It is important to note that highlighting consumer consciousness does not diminish the role of unconscious processes and the impact of sensory inputs on them. Our experience of the sensory world is delayed (see, Libet, 1999), which raises the possibility that all conscious mental functions are initiated unconsciously. Unconscious processes have been recognised as one of the primary causes of consumer behaviour (Williams and Poehlman, 2017; García-Madariaga et al., 2020; Ozkara and Bagozzi, 2021). The key point to discuss here is how a decrease and/or alteration of consciousness generated by a change in which sensory inputs are transmitted to consumers can strongly impact the way(s) in which they are persuaded, control themselves and make decisions (Baumeister et al., 2017; Plassmann and Mormann, 2017).

## The Challenge of Consumer Consciousness in XR

Not being able to smell perfume or touch clothes online prior to purchasing can be frustrating for consumers (Petit et al., 2019; Løkke-Andersen et al., 2021), which could also impact the extent to which they are conscious of their online experiences. Limiting sensory inputs online, at least relative to experiences in the physical world, could make the contents of online experience somehow less vivid for consumers, who would also be less alert (Nani et al., 2019). For example, Schlosser (2003) showed that when consumers interact with a product online (vs. passively view it), they engaged in greater mental imagery and had increased cognitive elaboration. Even if consumers do not have access to all of the sensory stimuli that are desirable for their decision-making, mental simulation can nevertheless still facilitate multisensory perceptual re-enactments (Petit et al., 2019; Elder and Krishna, 2021). For example, the visual exposure to appetising pictures of food not only activates regions of the visual cortex that represent object shape, it also activates gustatory areas in order to produce conceptual inferences about taste (Simmons et al., 2005). Thus, top-down visual imagery can affect information processing in the online environment in a way that is similar to bottom-up perceptual inputs, and thus help the consumer to be more conscious of their experiences (Spence et al., 2016).

It has been suggested that the richer the visual stimuli, the more vivid the resulting visual imagery (Marks, 1999; Basso et al., 2018). By including 3D-interactive views and virtual try-ons in online environments, marketers can facilitate the consumers’ visual imagery, in turn potentially filling-in some of the missing sensory inputs (Ho et al., 2013; Heller et al., 2019b). For example, Petit et al. (2021) highlighted how AR can facilitate the purchase intention for served (as compared to packaged) food by stimulating mental simulation of the eating process. These effects of visual-enabling technologies can now be reinforced by SETs (Velasco et al., 2018; Petit et al., 2019; Velasco and Obrist, 2020; Cornelio et al., 2021). For example, Heller et al. (2019a) reported that including touch control in an AR experience increased the consumer’s willingness to pay. By stimulating several of the senses during online experiences, consumer consciousness might thus be enhanced. However, the reality-virtuality continuum (Milgram and Kishino, 1994) integrates different experiences, merging real and virtual objects, which could confuse the consumer and thus adversely impact their consciousness.

**Figure 1:**
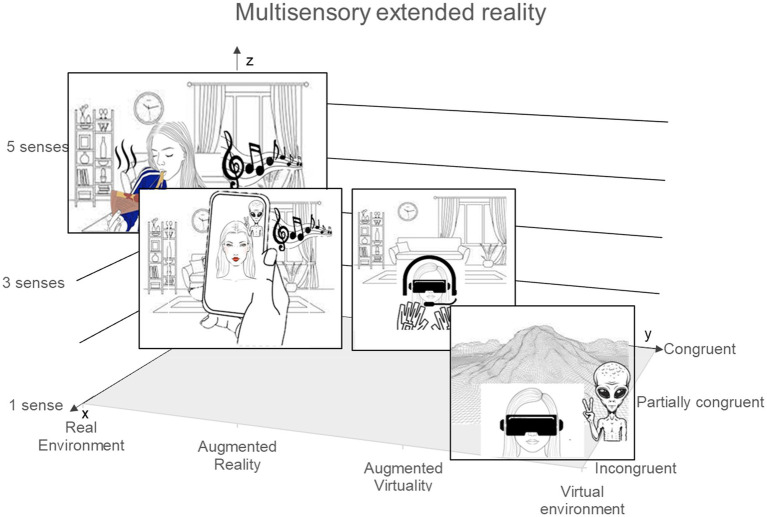
Multisensory XR including (1) the reality-virtuality continuum from Milgram and Kishino (1994), enhanced by (2) sensory inputs, and considering (3) their level of congruency with the environment. By considering this multisensory XR model, marketers can potentially stimulate one or more of the consumer’s senses (e.g. 1: vision, 3: vision, audition and touch, z-axis), from the real-life, real and virtual or only virtual environment (e.g. vision: virtual, audition: real, y-axis) and make these stimulations incongruent, partially congruent or completely congruent with the real-life environment and/or virtual environment (e.g. virtual alien through the smartphone in the physical living room with the noise of the television in the background, x-axis).

The reality-virtuality continuum ranges from real environments, through those that involve both real and digital elements (as in AR and augmented virtuality), to fully virtual ones (see Flavián et al., 2019). In this continuum, if the virtual elements were only visual, the SETs make it possible to expand them to other senses. Marketers could therefore potentially manipulate sensory stimuli from the real environment and/or use SETs to digitally stimulate the senses. They could also choose to stimulate only certain senses and choose whether the stimuli would be congruent with either the consumer’s physical environment (e.g. noise of the coffee machine in the consumer’s kitchen), the virtual environment (e.g. the noise of the sea on the beach), both (e.g. the smell of fruit juice) or completely incongruent (e.g. the sound of a cow mooing; see Figure 1). These differences in congruency can make mental simulation more-or-less difficult by affecting the ability to make predictions about the expected consumption experience based on the incoming sensory inputs. Future research is needed to understand the impact of these different multisensory extended reality (XR) experiences on consumer behaviour.

## Research Perspectives on Consumer Consciousness in XR

Our brain’s capacity to create a conscious representation of our bodily experience results from the complex interplay of perceptual streams involving, in particular, vision, touch and interoception (Maselli and Slater, 2013). Through the stimulation of the senses in XR, it is possible to provide consumers with the illusion of ownership over an artificial body (Maselli and Slater, 2013). This virtual body can potentially affect consumer consciousness and may exert a significant influence over their physiology and behaviour.

### Consumer Consciousness and the Illusion of Reality

Technologies have been used to create multisensory conflicts in order to manipulate self-consciousness (Ionta et al., 2011). For example, the ‘rubber hand illusion’ revealed that if participants observe a rubber hand being stroked synchronously with their own hand, they tend to report self-attribution of the rubber hand (Botvinick and Cohen, 1998; Ehrsson et al., 2004). An important aspect of such bodily self-consciousness is self-location (Arnold et al., 2017) and the sense of interoception (Ionta et al., 2011). New technologies such as VR have been shown to facilitate interoception and thus generate a body transfer illusion *via* the first-person perspective. Interestingly, depending on whether they are synchronised or not, visuotactile stimulation can lead to alterations of this first-person perspective and to the localization of the self-outside the body (Ionta et al., 2011). Thus, the congruent stimulation of multisensory stimuli can be used to create an illusion of reality, with positive effects on bodily self-consciousness.

If SETs can facilitate self-location in a virtual environment, can the same be said for the experiences that consumers might have with products? If these multisensory simulations match the brain’s expectations of what the consumption experience would actually be like in the real-world, the brain should treat the virtual products as more-or-less real (Gonzalez-Franco and Lanier, 2017). Experiencing such illusions might therefore be expected to promote the dematerialization of the consumers’ extended-self and by so doing increase their acceptance of digital products (Belk, 2013). The whole point is to generate perceptual representations of possible objects and/or environments in the user’s brain, by making them the probable causes of a sensory signal. Ideally, future research will identify which particular combinations of stimuli (sensory−/multisensory and digital/physical) best promote the illusion that the product is real and favour positive evaluations.

### Consumer Consciousness and Impossible Experiences

Velasco et al. (2021a) introduced a model to think about impossible experiences in XR. This reality-impossibility model includes two continua, namely, the reality-fantasy character of objects and environments, and the extent to which they follow the laws of physics-other laws. Acceptance of such impossible experiences can lead marketers to invent products and uses that would simply not be possible in the real-world. For example, one can imagine that consumers might be provided with a means of chatting with friends by appearing on the back of a unicorn. However, creating an illusion of reality for this kind of experience may well be difficult to achieve. There is a risk that the brain would simply reject the illusion, if the discordance between afferent sensory inputs and the intended state becomes too great (Gonzalez-Franco et al., 2010), which could make consumers hesitant to act (Haggard et al., 2002). Future research aims to determine whether the illusion is easier to create/maintain if sensory inputs are consistent with the consumer’s physical environment (e.g. the noise of the coffee machine in the kitchen) and/or with virtual objects (e.g. the sound of the unicorn’s wings flapping).

Through impossible experiences in XR, brands could thus obtain new means of communication and experience design. Metaphors have been used to describe things that do not exist or that are physically impossible to do (Gibbs and Matlock, 2008). In marketing, metaphors succeed in creating associations between brands and concepts (Cornelissen, 2003; Hirschman, 2007). If instead of getting consumers to imagine themselves having wings, Red Bull gave them wings in XR, how would that impact on their behaviour/beliefs? Future studies should try to understand the effect of such experiences on consumer behaviour.

### Avatar Consciousness

XR technologies can produce a full-body illusion *via* avatars (Kokkinara and Slater, 2014; Gonzalez-Franco and Lanier, 2017), and they might allow consumers to experience the same sensations of ownership over a virtual body. The sense of embodiment has been shown to be closely related to the sense of self (Cassam, 2012). Thus, the re-embodiment of consumers in the body of an avatar can impact their self-perception (Scholz and Duffy, 2018). For example, viewing oneself in an AR mirror can reduce the ideal-actual attractiveness gap (Javornik et al., 2021). Consumers tend to create avatars similar to their real selves, but in a more attractive version, which affects their online behavioural traits (Messinger et al., 2019). Sometimes avatars can be very distant from the real person, which can lead to subsequent changes in behaviour. For instance, an improvement of negotiation skills has been observed when a subject is embodied in a taller avatar (Yee and Bailenson, 2007). In terms of consumer behaviour, this can lead individuals to purchase those products that might match their avatar’s bodily form but not necessarily their own. It is important that marketing research questions how, thanks to SETs, to stimulate the perceptual system to facilitate, in some cases, the awareness of one’s body, and in the other, the embedding in an avatar body.

### When Is Consumer Consciousness in XR Relevant?

SETs provide new information to users that could override direct access to their bodily sensations (Eichenberg and Wolters, 2012). Research on pain has shown that the sight of a painful part of one’s body in VR may lead to a reduction of activity in the somatosensory cortex (Matamala-Gomez et al., 2019). This manipulation of the body image (e.g. size and colour) reduces the perception of pain (Moseley et al., 2008; Martini et al., 2013). This type of research could be interesting in consumer behaviour, particularly in the context of public health. For example, Narumi et al. (2012) used AR to create the illusion that the cookie that people were eating was actually larger than, in fact, was the case, leading them to reduce their immediate consumption. It can be interesting to explore whether consumers can more easily maintain their diet by consuming healthy foods and/or reducing their portion size if they happen to be eaten by their virtual body rather than by them directly (one might also consider the large portions of energy-dense foods for broadcast jockeys in Mukbang videos; see Spence, 2017). It might also be interesting to investigate how the manipulation of the virtual body (e.g. shape, size and colour) can affect such behaviours (Matsangidou et al., 2017; Narumi, 2021).

## Discussion

The way in which marketers stimulate the consumer’s senses undoubtedly plays an essential role in determining how sensory information will be consciously perceived by the latter. Consciousness is important for consumers to make those decisions that best fit their enlightened self-interest (Baumeister et al., 2017). However, the lack of varied sensory inputs, relative to the physical world, in our online experiences has long been a limitation to consumer consciousness. SETs provide different ways of stimulating the consumer’s senses, whether by integrating sensory stimuli from the physical world into a virtual one, by integrating digital sensory stimuli into the physical world, or else by combining them. Marketers can also choose which senses to stimulate, and if they want to make these stimulations congruent with the consumer’s physical and/or digital environment, or even to make them completely incongruent.

Future research is needed in order to understand how best to stimulate the consumer’s senses to adapt to different contexts and make them more conscious of their experiences. XR *via* external physiological sensors (e.g. heart rate) might also help to improve this understanding (Siriborvornratanakul, 2016). Beyond technical challenges, stimulating multiple senses in XR also bring about ethical questions (Gallace et al., 2012). They may increase the problem of addiction to social media and have long-term impact on consumer’s mental and physical health (Madary and Metzinger, 2016; Winn, 2021). Using photo filters on Instagram or Snapchat has been shown to impact self-acceptance and wellbeing (Javornik et al., 2022), leading some people to turn to plastic surgery (Murphy-Kelly, 2020). Furthermore, a woman reported having been sexually harassed on Meta’s platform (Basu, 2021). There is a distinction between realism in the physical (appearance of the virtual features) versus psychological (sensation that what happens virtually) realm (Slater et al., 2020), and marketers need to ensure that both are positive for consumers.

To conclude, the multiple ways of stimulating the consumer’s senses in XR are potentially disruptive for the latter in terms of impacting their consciousness. The metaverse will provide marketers with the possibility of creating virtual products and imagining impossible experiences for the avatars of consumers. How, exactly, the senses will be stimulated will depend on how consumers will be conscious of their body and of their surroundings that will certainly affect the success of their marketing experiences. It is therefore fundamental that marketers wonder about the type of multisensory mixed reality they wish to create.

## Data Availability Statement

The original contributions presented in the study are included in the article/supplementary material; further inquiries can be directed to the corresponding author.

## Author Contributions

All authors listed have made a substantial, direct, and intellectual contribution to the work.

## Conflict of Interest

The authors declare that the research was conducted in the absence of any commercial or financial relationships that could be construed as a potential conflict of interest.

## Publisher’s Note

All claims expressed in this article are solely those of the authors and do not necessarily represent those of their affiliated organizations, or those of the publisher, the editors and the reviewers. Any product that may be evaluated in this article, or claim that may be made by its manufacturer, is not guaranteed or endorsed by the publisher.
